# Supplementary material for: Liposomal Nanovaccine Containing α-Galactosylceramide and Ganglioside GM3 Stimulates Robust CD8+ T Cell Responses via CD169+ Macrophages and cDC1
Source: Vaccines (Basel). 2021 Jan 16;9(1):56. doi: 10.3390/vaccines9010056 (PMC7830461; doi:10.3390/vaccines9010056)
Supplement: Supplementary file 1 [file vaccines-09-00056-s001.pdf]

vaccines

MDPI

Article

Liposomal Nanovaccine Containing α-Galactosylceramide and Ganglioside GM3 Stimulates Robust CD8⁺ T Cell Responses via CD169⁺ Macrophages and cDC1

Joanna Grabowska [1,†], Dorian A. Stolk [1,†], Maarten K. Nijen Twilhaar [1], Martino Ambrosini [1], Gert Storm [2,3,4], Hans J. van der Vliet [5,6], Tanja D. de Gruijl [5], Yvette van Kooyk [1] and Joke M.M. den Haan [1,*]

1 Department of Molecular Cell Biology and Immunology, Amsterdam UMC, Cancer Center Amsterdam, Amsterdam Infection and Immunity Institute, Vrije Universiteit Amsterdam, 1081 HZ Amsterdam, The Netherlands; d.stolk@amsterdamumc.nl (D.A.S.); m.nijentwilhaar@amsterdamumc.nl (M.K.N.T.); ambrosinimartino78@gmail.com (M.A.); y.vankooyk@amsterdamumc.nl (Y.v.K.); j.denhaan@amsterdamumc.nl (J.M.M.d.H.)

2 Department of Pharmaceutics, Utrecht Institute for Pharmaceutical Sciences, Utrecht University, 3584 CG Utrecht, The Netherlands; G.Storm@uu.nl

3 Department of Biomaterials Science and Technology, University of Twente, 7500 AE Enschede, The Netherlands

4 Department of Surgery, Yong Loo Lin School of Medicine, National University of Singapore, 119228, Singapore

5 Department of Medical Oncology, Amsterdam UMC, Cancer Center Amsterdam, Amsterdam Infection and Immunity Institute, Vrije Universiteit Amsterdam, 1081 HV Amsterdam, The Netherlands; jj.vandervliet@amsterdamumc.nl (H.J.v.d.V.); td.degruijl@amsterdamumc.nl (T.D.d.G.)

6 Lava Therapeutics, 3584 CM Utrecht, The Netherlands

* Correspondence: j.denhaan@amsterdamumc.nl; Tel.: +31-20-4448080

† Authors contributed equally.

Citation: Grabowska, J.; Stolk, D.A.; Nijen Twilhaar, M.K.; Ambrosini, M.; Storm, G.; van der Vliet, H.J..; de Gruijl, T.D.;van Kooyk, Y..; den Haan, J.M.M.. Liposomal nanovaccine containing α-galactosylceramide and ganglioside GM3 stimulates robust CD8+ T cell responses via CD169+ macrophages and cDC1. *Vaccines* **2021**, *9*, 56. https://doi.org/10.3390/vaccines9010056

Received: 16 December 2020
Accepted: 10 January 2021
Published: 16 January 2021

Publisher's Note: MDPI stays neutral with regard to jurisdictional claims in published maps and institutional affiliations.

Copyright: © 2021 by the authors. Submitted for possible open access publication under the terms and conditions of the Creative Commons Attribution (CC BY) license (http://creativecommons.org/licenses/by/4.0/).

Supplementary Materials: The following are available online at www.mdpi.com/xxx/s1, Figure S1:Gating strategy used for analysis of flow cytometry data. S2: Red pulp macrophages, cDC1 and B cells exhibit minimal liposome uptake. Figure S3: CD169⁺ macrophages, red pulp macrophages, DCs and B cells express CD1d on their surface. Figures S4: Treatment with DT depletes CD169-expressing macrophages in CD169-DTR mice. Figure S5: cDC1 are depleted in Batf3KO animals. Figure S6: GM3-αGC liposomes trigger NKT cell-mediated DC maturation and NK cell activation 16h p.i.

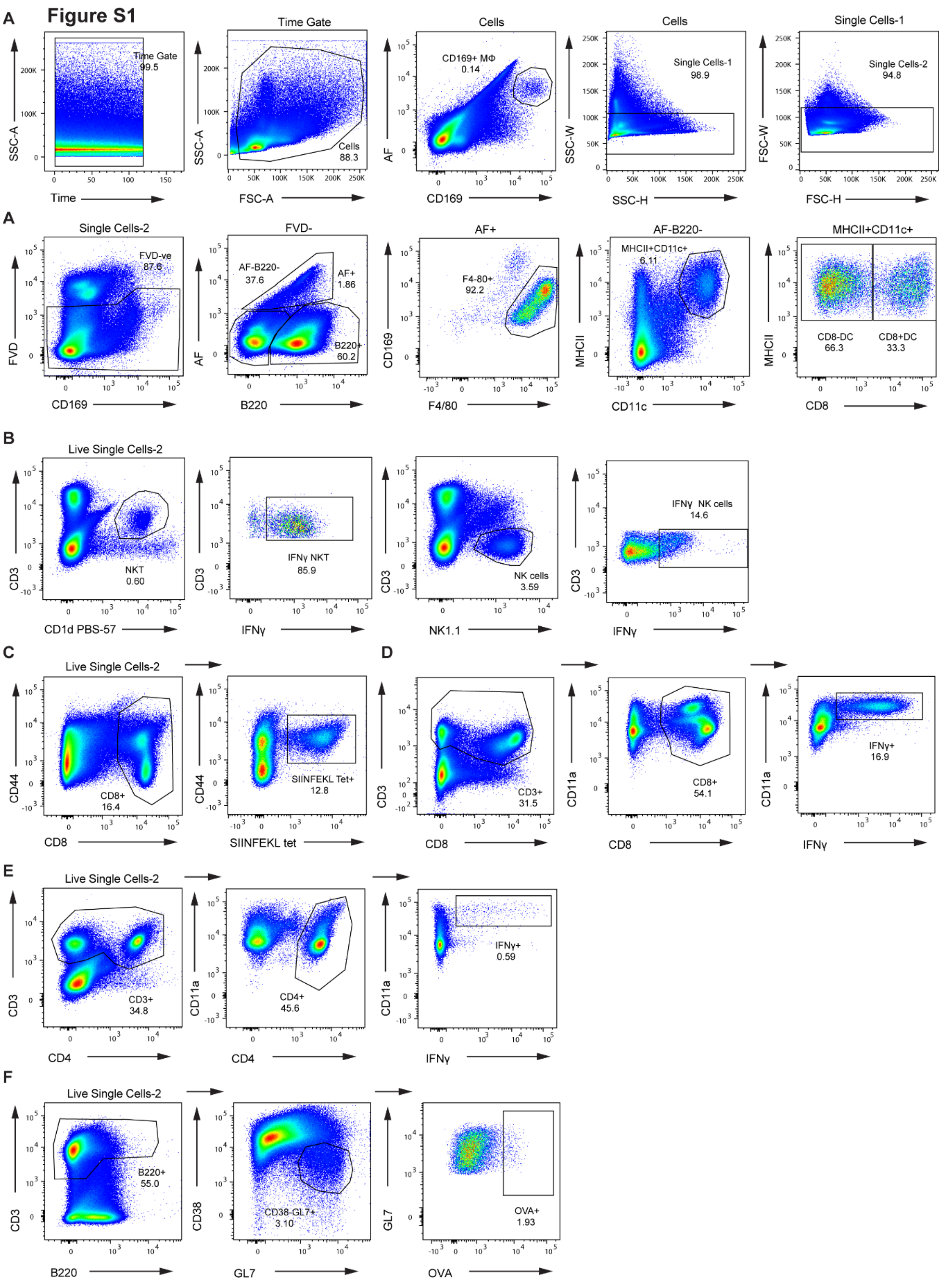

Figure Supplementary 1. Gating strategy used for analysis of flow cytometry data. (A) Gating strategy employed to identify CD169⁺ macrophages, red pulp macrophages (AF⁺F4/80⁺), B cells (B220⁺) and DCs (MHCII⁺CD11c⁺CD8⁺/⁻). (B) Gating strategy employed to identify NKT (CD1d PBS-57⁺CD3^{int}) and NK cells (CD3⁻NK1.1⁺). (C-E) Gating strategy employed to identify SIINFEKL⁺ CD8⁺ T cells (C), IFNγ-producing CD8⁺ T cells (D) and CD4⁺ T cells (E) and OVA⁺ germinal center B cells.

Figure S2

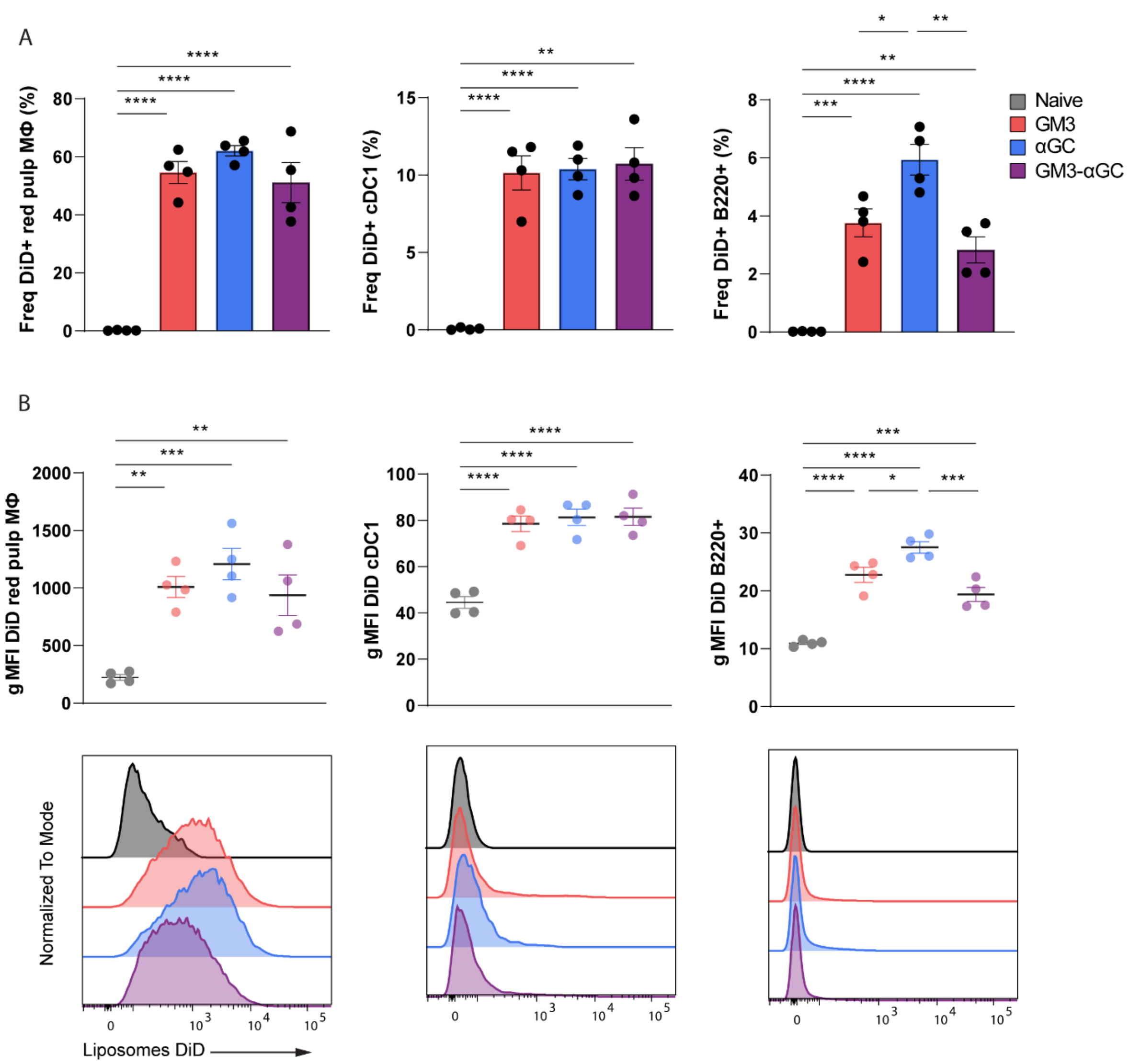

Figure Supplementary 2. Red pulp macrophages, cDC1 and B cells exhibit marginal liposome uptake. (A-B) Liposome uptake at 2h p.i. by red pulp macrophages, cDC1 and B cells from WT mice illustrated by (A) frequency of DiD⁺ cells and (B) DiD fluorescence signal (gMFI; upper panels) and histogram overlays (lower panels) determined by flow cytometry. Data are mean ± SEM from one experiment, each symbol represents one mouse (one-way ANOVA with Tukey's test: *$p < 0.05$, **$p < 0.01$, ***$p < 0.001$, ****$p < 0.0001$).

Figure S3

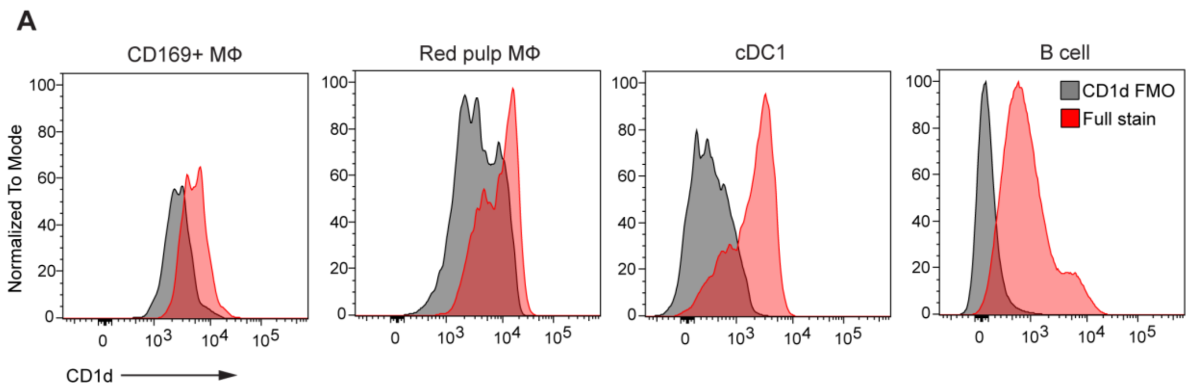

Figure Supplementary 3. CD169⁺ macrophages, red pulp macrophages, DCs and B cells express CD1d on their surface. Representative histogram overlays showing expression of CD1d per cell population, determined by flow cytometry. Black peaks illustrate CD1d FMO (fluorescence minus one) control and red peaks illustrate fully stained sample.

Figure S4

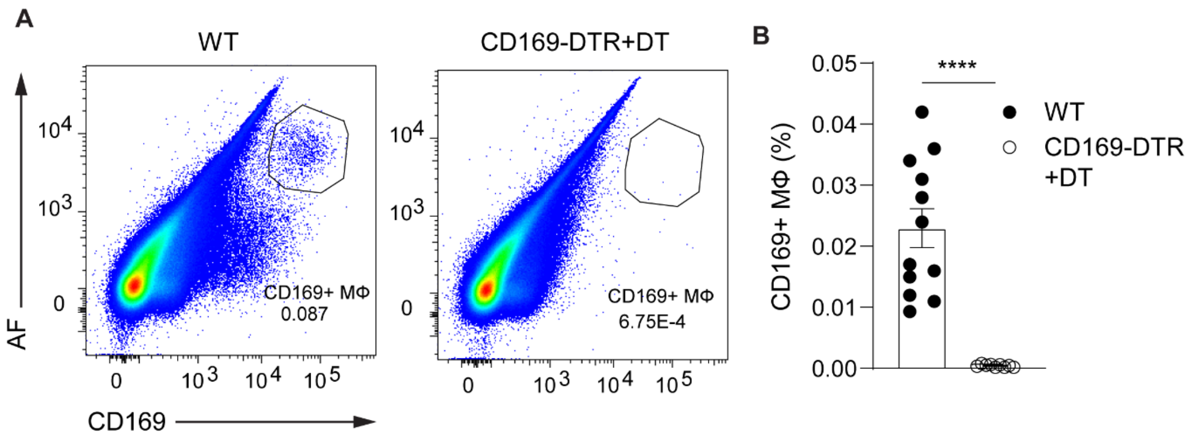

Figure Supplementary 4. Treatment with DT depletes CD169-expressing macrophages in CD169-DTR mice. (A) Representative flow cytometry plots of showing CD169^{+} macrophages in WT and DT-treated CD169-DTR mice. DT, diphtheria toxin (B) Quantification of the CD169^{+} macrophage population in WT and DT-treated CD169-DTR mice. Data are mean ± SEM from one experiment, each symbol represents one mouse (one-way ANOVA with Tukey's test: *p < 0.05, **p < 0.01, ***p < 0.001,****p < 0.0001).

Figure S5

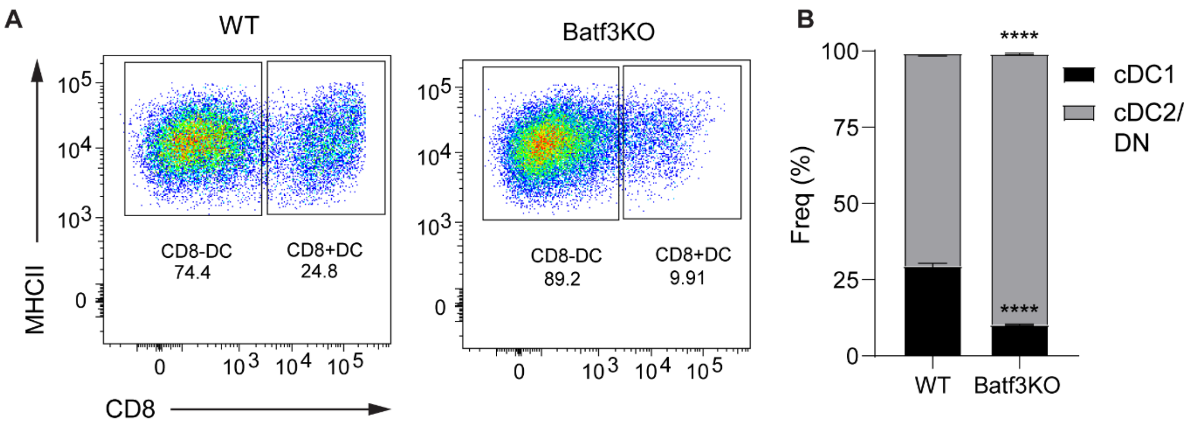

Figure Supplementary 5. cDC1 are depleted in Batf3KO animals. (A) Representative flow cytometry plots of showing cDC1 (gated on MHCII^{+}CD11c^{+}) in WT and Batf3KO mice. (B) Quantification of the frequency of cDC1 in WT and Batf3KO mice. Data are mean ± SEM from one experiment, each symbol represents one mouse (one-way ANOVA with Tukey's test: *p < 0.05, **p < 0.01, ***p < 0.001,****p < 0.0001).

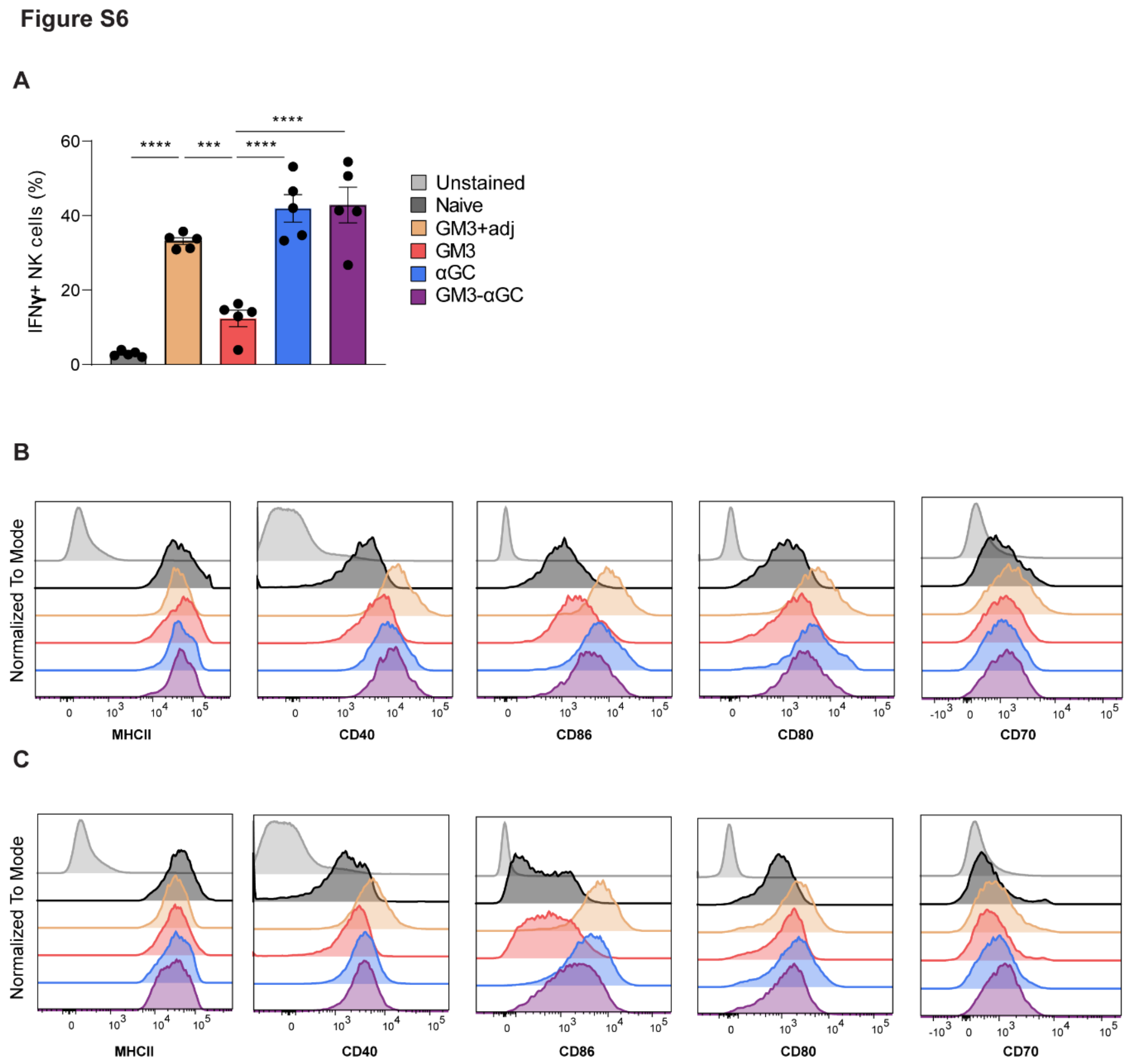

Figure Supplementary 6. GM3-αGC liposomes trigger NKT cell-mediated DC maturation and NK cell activation 16h p.i.. GM3+adj represents GM3 liposome co-injected i.v. with αCD40/poly I:C. (A) Activation of NK cells illustrated by frequency and IFNγ production, determined by flow cytometry. (B-C) Representative histogram overlays showing expression of MHCII, CD40, CD86, CD80 and CD70 on cDC1 (B) and cDC2 (C) at 16h p.i. with liposomes, determined by flow cytometry. Data are mean ± SEM from one experiment, each symbol represents one mouse (one-way ANOVA with Tukey's test: *p < 0.05, **p < 0.01, ***p < 0.001,****p < 0.0001).
